# Introduction to the principles and methods underlying the recovery of metagenome‐assembled genomes from metagenomic data

**DOI:** 10.1002/mbo3.1298

**Published:** 2022-06-09

**Authors:** Gleb Goussarov, Mohamed Mysara, Peter Vandamme, Rob Van Houdt

**Affiliations:** ^1^ Microbiology Unit, Belgian Nuclear Research Centre (SCK CEN) Mol Belgium; ^2^ Laboratory of Microbiology and BCCM/LMG Bacteria Collection, Faculty of Sciences Ghent University Ghent Belgium

**Keywords:** annotation, assembly, binning, metagenome‐assembled genome, metagenomics, sequencing

## Abstract

The rise of metagenomics offers a leap forward for understanding the genetic diversity of microorganisms in many different complex environments by providing a platform that can identify potentially unlimited numbers of known and novel microorganisms. As such, it is impossible to imagine new major initiatives without metagenomics. Nevertheless, it represents a relatively new discipline with various levels of complexity and demands on bioinformatics. The underlying principles and methods used in metagenomics are often seen as common knowledge and often not detailed or fragmented. Therefore, we reviewed these to guide microbiologists in taking the first steps into metagenomics. We specifically focus on a workflow aimed at reconstructing individual genomes, that is, metagenome‐assembled genomes, integrating DNA sequencing, assembly, binning, identification and annotation.

## Graphical abstract

Metagenomics greatly facilitates the identification and characterization of known and novel microorganisms. The plethora of available bioinformatics tools with various levels of complexity rely on (shared) fundamental principles and methods, which are often not detailed or scattered. Here, we review these principles and methods with a focus on reconstructing individual genomes from metagenomics data by integrating DNA sequencing, assembly, binning, identification and annotation.

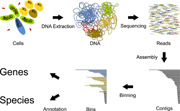

## 1 FROM DNA TO METAGENOME‐ASSEMBLED GENOMES (MAGs)

**Figure 1:**
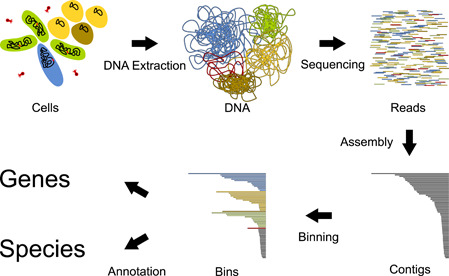
Metagenome analysis scheme. First, DNA in the test sample is extracted. Then, reads are produced by sequencing, exposing the DNA's sequence as a series of fragments. After this, overlapping reads are assembled, producing “contigs.” During these two steps, the source of each sequence is unknown; therefore, an additional separation step called binning is necessary. Finally, each sequence needs to be annotated, which is the process of assigning meaningful names to different subsequences.

The potential of metagenomics to explore and study new environments will become fundamental in the coming decade. Thanks to the ability to bypass the labor‐intensive isolation and cultivation steps, this approach could theoretically be used to detect and characterize a much wider range of prokaryotes. However, metagenomics is a broad term that encompasses different types of analyses, with various levels of complexity. Furthermore, the underlying principles and techniques are often seen as common knowledge and either not detailed or fragmented. Therefore, we reviewed these to guide microbiologists in taking the first steps into metagenomics. We specifically focus on a workflow aimed at reconstructing individual genomes in a sample, integrating DNA sequencing, assembly, binning, identification and annotation (Figure 1).

## 2 DNA SEQUENCING

A few steps precede the actual DNA sequencing, that is, sample collection and storage, and DNA extraction and purification. Although out of the scope, it is important to highlight the importance of these steps (Pollock et al., 2018). In particular, care should be taken to avoid contamination and biases due to DNA extraction and degradation during processing and storage (Han et al., 2019; Nahar et al., 2021; Sar et al., 2018). Comparative studies indicated that the choice of DNA extraction method affects the outcome, with mechanical and/or enzymatic pretreatments being often superior (Gryp et al., 2020; Henderson et al., 2013). Furthermore, with long‐read sequencing emerging, extracting DNA of sufficient molecular weight, purity and quantity becomes even more critical (Maghini et al., 2021), and is not always unbiased for real samples (Bickhart et al., 2022; Moss et al., 2020). Finally, in cases where the total amount of DNA is low, an optional amplification step, such as multiple displacement amplification or linear amplification, may be performed as well (Bowers et al., 2015), which may lead to additional biases. Indeed, in some aqueous environments, despite sampling hundreds of liters of water, only picograms or nanograms can be extracted, as opposed to the micrograms needed for amplification‐free high‐quality metagenome sequencing.

**Figure 2:**
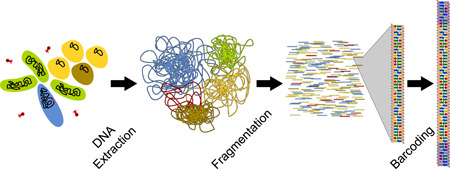
A metagenomic community contains multiple strains with varying abundance and is also likely to contain viruses and eukaryotic cells. The first step is to extract the genetic material (DNA or RNA) by removing other organic and inorganic molecules. Next, sequencing techniques require the DNA to be fragmented down to a length that the machine can process. In addition, when multiple samples are sequenced in the same run, a “barcode” sequence has to be added to each read to determine the sample from which it originated. These steps are common to all sequencing approaches, whereas further steps are technology‐specific.

Once DNA is extracted and purified, the next step is library preparation. This process may be split up into a number of substeps (Head et al., 2014). Some may be optional depending on the sequencing approach. The first step is fragmentation (physical or enzymatic fragmentation) and/or size selection (Figure 2), in which DNA fragments of the desired read length are enriched. For approaches that rely on Illumina paired‐end sequencers (see below), this step is important since only the ends of each fragment are sequenced. For long‐read sequencing, fragmentation can potentially help to increase throughput by ensuring that more fragments can be sequenced completely and size selection can be performed to get rid of shorter fragments. The second step is essentially a finalization step that needs to be performed after fragmentation, in which the fragments are altered in a way to make them amenable to further processing. Typically, the aim is to ensure that the resulting fragments are stable double‐stranded DNA. At this point, fragments may be labeled with “barcodes” for cases where multiple samples are analyzed at once, which is followed by a final step where additional molecules are bound that allow the various sequencing technologies to function.

Metagenome sequencing requires extremely high throughput to capture the full genomes of a large number of diverse bacteria present in samples. As such, only second‐ and third‐generation high‐throughput sequencing methods can be used. Currently, three major families of sequencing platforms are suitable for this purpose.

### 2.1 Sequencing by synthesis

**Figure 3:**
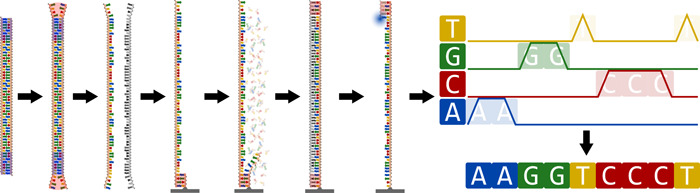
Illumina sequencing. The adapters for Illumina sequencing have mismatch sequences to enable easier binding of DNA to the substrate used for sequencing, to which only one strand is bound. The complementary sequence of the said strand is then produced through a polymerase chain reaction. Next, the original strand is detached and washed away, and actual sequencing begins by successive cycles of binding complementary nucleotides, recording fluorescent signals and washing the fluorescent dyes for the next cycle to be possible. The operation is terminated after a fixed number of cycles.

The best‐known are the Illumina (previously Solexa) sequencers, including the MiSeq, HiSeq, NovaSeq and NextSeq series of sequencers, which produce short reads (100−300 bases) with low error rates (<1%). Although variants of the technique are used, including single‐end and mate‐pair sequencing, paired‐end sequencing in which reads represent the ends of DNA fragments with a size specified by the library preparation step is most common in metagenomics applications (Figure 3). Although the read length of Illumina machines depends on the number of cycles that are performed, the length of DNA fragments is important as well. If the fragments are sufficiently short, the ends will overlap, which provides assemblers with an easy way to transform the reads into longer DNA sequences. However, longer fragment lengths have the potential to result in less fragmented genomes, and as such, insert size (between sequencing adapters) should be chosen carefully (Cho et al., 2016).

An alternative to Illumina is BGI's DNBseq technology. The latter operates on the same principle, except that “DNA nanoballs” are used instead of lanes, which allow a more consistent signal for each sequence at similar costs at the time it was first proposed (Bonetta, 2010; Drmanac et al., 2010). According to H.‐M. Kim et al. (2021), the current DNBseq‐G50 (BGIseq‐500) platform is comparable to the Illumina HiSeq 2500 in both accuracy and throughput. Although the HiSeq 2500 is not the most performant Illumina sequencer, this shows that the technologies have comparable output from the point of view of bioinformatics. As the technology is much more recent than Illumina sequencing, having been officially released only in 2017, it has yet to catch up to Illumina's popularity, but its lower cost will likely ensure a rise in popularity in the coming years.

Other sequencing‐by‐synthesis methods, such as Ion Torrent and pyrosequencing (also commonly referred to as 454‐sequencing), exist but have mostly been replaced by those mentioned above. This is likely due to their issues with identifying homopolymeric repeats and lower throughput (Balzer et al., 2010; Bragg et al., 2013; C. Luo, Tsementzi, et al., 2012).

Next to the standard output, Illumina also developed synthetic long‐read technology, called TruSeq synthetic long‐reads or TSLR (McCoy et al., 2014). However, because that approach utilizes well plates rather than microbeads, the number of barcodes that can be used for that approach is more limited, making the recovery of fragments more computationally challenging. Similarly, BGI has also introduced “single‐tube long fragment read” (stLFR) sequencing, which is an alternative to the standard library preparation methods and can be used to effectively produce long reads from short reads at a reduced cost (O. Wang et al., 2019). The general principle of the approach is to add identical barcodes to reads produced from the same long fragment, with each barcode being unique to a given fragment.

### 2.2 Pacific Biosciences “single‐molecule real‐time sequencing”

**Figure 4:**
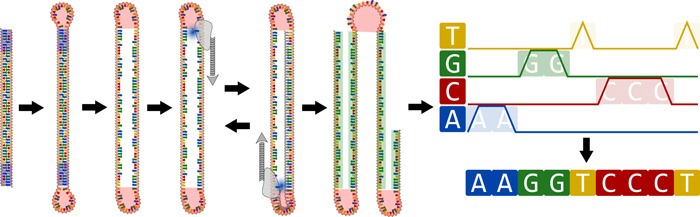
PacBio Sequencing adapters enable the circularization of DNA fragments. Next, a polymerase reads the circularized fragment repeatedly, producing a continuous string of fluorescent signals containing the forward and reverse strands and adapter sequences. Adapter removal splits the sequence into “sub‐reads” (in green), which can then be combined to create a high‐quality read by a process called circular consensus.

The Pacific Biosciences (PacBio) sequencers, including the RS, RS II, Sequel and Sequel II sequencers, use adapters to circularize DNA fragments, which are then placed inside wells where a polymerase copies the fragment using labeled nucleotides, producing a detectable fluorescent signal when they are bound to their complement by the polymerase. As each well should only contain one fragment and each nucleotide is labeled with a different fluorescent molecule, the polymerization process produces a continuous signal that represents the fragment being sequenced. Because the fragments are circularized, short fragments may be read multiple times before the polymerization process terminates, whereas long fragments may sometimes fail to be sequenced fully (Figure 4). As a result, two strategies can be used with this technology.

In one approach, DNA is fragmented into shorter sequences of a length varying between 1,000 and 15,000 bases. Because these fragments are relatively short, the process described above can read the same sequence multiple times, with each pass of the polymerase producing a “subread” (Figure 4). This produces highly accurate “circular consensus sequences” (CCSs), as each base is sequenced multiple times. One of the early studies on this approach concluded that 50% of the reads could be made to be 99.9% accurate with just four subreads (Travers et al., 2010), although the final CCSs were mostly shorter than 2 kb (Hebert et al., 2018; Travers et al., 2010). With improvements in chemistry primarily increasing the longevity of the polymerase, longer CCS reads of around 15 kb could be obtained while maintaining accuracy (99.9% accuracy with 10 passes) and without the need for a substantial increase in the quality of individual subreads (Wenger et al., 2019).

In the other approach, fragmentation results in lengths of around 50 kb. This produces a much lower accuracy (an error rate of 10%−15%) when compared to CCS, but the reads are also longer and can cover larger portions of the target genome. Since the number of reads produced in a run is limited by the sequencer, this effectively also increases throughput.

### 2.3 Oxford Nanopore Technologies “ONT sequencing”

**Figure 5:**
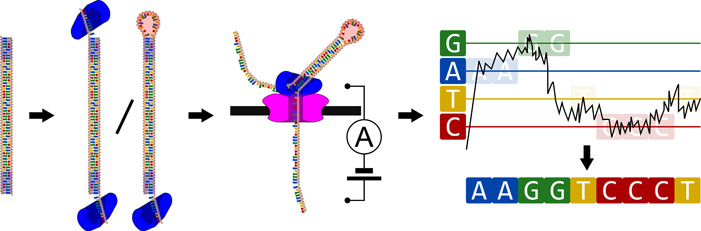
ONT sequencing adapters are added with a “motor” enzyme (blue), which serves to control the speed at which DNA passes through pores, either on both ends of the DNA fragments in the case of one‐dimensional reads or combined with a hairpin adapter in the case of two‐dimensional reads. The motor enzyme attaches porins (purple) embedded in a thin membrane, then progressively pushes ssDNA through it. As it does, the ionic current passing between the sides of the membrane changes depending on the nature of the nucleotides that occupy the most narrow region of the porin.

The Oxford Nanopore Technologies (ONT) sequencers, like the PacBio sequencers, operate in real time and are capable of producing the longest reads out of the three technologies. These instruments reconstruct DNA sequences based on current fluctuations elicited by DNA molecules when they pass through nanopores embedded in a membrane (Deamer et al., 2016; Jain et al., 2016; Rang et al., 2018) (Figure 5). This approach relies on a one‐dimensional signal and the interpretation of that signal is not as straightforward as the detections of four separate wavelengths associated with each type of nucleotide in fluorescence‐based techniques. Moreover, due to the nanopore size, it is not directly possible to measure individual bases, but rather the combined effect of the bases that occupy the most narrow point of the nanopore is what is measured, which depends on the type of nanopore being utilized. As a result, this sequencing method requires a more complex method to transform the raw sequencer signal into usable reads when compared to other platforms. Because ONT is the most recent of the three families of sequencers presented here, with the first commercial model formally released in 2015, it is difficult to gauge the accuracy of this method, as some of the errors may be due to not fully mature base‐calling software. Currently, the accuracy of base‐called reads lies around 90% (Wick et al., 2019). However, this may depend on the organism, as shown by Krishnakumar et al. (2018), where the reads of three species had average identity scores of 81.3%, 86.2%, and 89.2%, respectively. It should be noted that these numbers pertain to so‐called 1D reads, which are commonly used due to their ease of implementation. By contrast, there also exist 1D2 and 2D reads, which are more accurate but tend to have lower throughput and length (Weirather et al., 2017). Moreover, the latest R10 chemistry promises to improve these figures to above 97%, and this trend is likely to continue in the future as ONT have consistently been improving the performance of their sequencers and preparation kits (Amarasinghe et al., 2020). Until such high‐quality reads become available, the error rate of ONT sequencing precludes them from being used without an assembly step, that is, the useful output from ONT must necessarily be derived from multiple distinct DNA molecules rather than being useful as individual reads. In addition, having an assembly is required for polishing tools such as medaka (Oxford Nanopore Technologies, 2018).

## 3 ASSEMBLY

Although some reads can be used directly, this may not always be sufficient, as they are either too short or too inaccurate for applications such as closing genomes or detecting SNPs, respectively. Therefore, the next important step in (meta‐)genome analysis is assembly, in which reads are assembled into longer, contiguous sequences (contigs). Ideally, contigs should correspond to individual replicons, but this is rarely the case. It is also important to mention that in metagenomics the sequenced DNA fragments originate from different cells and include potential differences. As such, genomes assembled from metagenomes (MAGs) produced with current technology cannot be obtained directly after an assembly step, and are derived from the pan‐genome of taxonomically related groups within the target environment rather than individual genomes.

There are currently two dominant approaches to performing assembly. The first, and older, approach is called overlap, layout, consensus (OLC), whereas the newer approach is de Bruijn graph‐based (DBG) (Flicek & Birney, 2009; Z. Li et al., 2012; Miller et al., 2010; Schatz et al., 2010). OLC consists, as its name suggests, of three steps. During the first step, all reads are compared to each other to identify a possible overlap. This produces a graph with many redundant paths. The next step (layout) simplifies this graph to remove the redundant paths, so that the reads may be ordered in relation to each other. The final step consists of defining the consensus sequence from the reads that cover it. The main disadvantage of this method is speed, as both the overlap and consensus steps require computationally expensive alignment.

DBG functions in a different manner, as it was proposed to bypass the overlapping step, which rendered OLC unusable for the kind of data produced by second‐generation sequencers such as those in the Illumina family of sequencers. Rather than building a graph based on overlaps, DBG uses overlapping oligonucleotides of fixed size (often called *k*‐mers) as the basis for building a graph, thereby avoiding explicit alignment. This graph can then be traversed to uncover the original sequence. Although it removed the need to explicitly align sequences, the DBG approach is more sensitive to sequencing errors and has trouble resolving repetitive regions. However, neither of these drawbacks is particularly relevant for Illumina short reads, which have high accuracy, and whose length is also insufficient to resolve long repeats.

As third‐generation sequencing (including ONT and PacBio) is becoming more common, and available computational resources are increasing, OLC assemblers are once again gaining importance thanks to their ability to resolve repeats. However, because of this recent paradigm change, the state‐of‐the‐art in assembly software is evolving rapidly. New more performant tools that can handle multiple types of reads, either separately or together as “hybrid” assemblers, are being published frequently.

### 3.1 OLC assemblers

**Figure 6:**
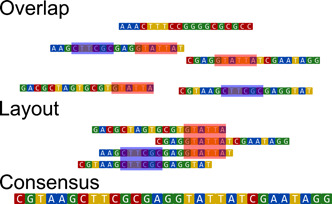
The Overlap−Layout−Consensus scheme follows a three‐step approach, whereby the sequenced reads are first parsed for potential overlapping sequences, after which the layout step determines their relative position, and the consensus steps determine the final sequence.

“Overlap−layout−consensus” (OLC) assemblers, which are conceptually more straightforward than the DBG assemblers, are separated into three steps (Figure 6), each with a specific purpose (Paszkiewicz & Studholme, 2010). At its core, the OLC approach relies on finding which reads align with each other, and how. Overlapping reads can then be combined into longer sequences, and by repeating this process, eventually, reconstruct the genome from which the reads originated. One thing to note here is that OLC assemblers are distinct from greedy extension assemblers (Miller et al., 2010), which perform this step by using only one read.

In the first—overlapping—step, matching fragments are identified. There are ways to determine whether two reads have the potential to contain alignments rapidly, including for example whether or not they contain identical subsequences (Pop, 2009). This can help to reduce the computational time necessary to align every single read against every other read, and is in part why OLC assemblers perform so well with long reads, as the cost of a single alignment is reduced considerably and the number of alignments is comparatively low. Conversely, the overlapping step is particularly costly for short high‐throughput reads, as the cost of each alignment is not that much larger than checking whether reads have the potential to align, whereas the number of such alignments can be very large. The results of the overlapping step can be summarized as a graph that contains the relative position of each read to the reads that share an alignment with it. The next—layout—step consists of the construction and analysis of this graph (Miller et al., 2010). Finally, not all reads will be concordant due to sequencing errors, and an additional—consensus—step is necessary to resolve such issues. This step can also be computationally costly, as it requires accurate multiple sequence alignments (MSA).

Unlike DBG assemblers, OLC assemblers are not constrained by a fixed *k*‐mer length, and can therefore be more flexible and accurate, especially for long reads, because they use the full length of the supplied reads rather than splittng them further. The main disadvantage of OLC assemblers is their speed. Although heuristic methods have been developed, which can drastically reduce the computational costs of alignment (see below), these remain slower than the DBG approach.

In the early 2000s, a number of OLC assemblers were developed, which have for the most part become irrelevant due to lacking the optimizations necessary to make these assemblers usable in a modern context. Notable among these was the Celera assembler (Myers et al., 2000), which was used as the basis for the CABOG assembler (Miller et al., 2008) and later for CANU (Koren et al., 2017; Nurk et al., 2020). Having such a long development history (by the standards of modern assemblers), CANU is complex yet still relatively fast by comparison to its predecessor. Other OLC assemblers, such as Shasta (Shafin et al., 2020), RedBean/WTDBG2 (Ruan & Li, 2020) and MetaFlye (Kolmogorov et al., 2020), are faster than CANU, but also less accurate for metagenome analysis (Wick & Holt, 2019).

### 3.2 DBG assemblers

**Figure 7:**
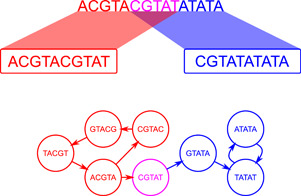
A de Bruijn graph in the context of genomics is a representation of sequences as a graph of short(er) oligonucleotides, which only differ by one position. Here, a “true” sequence produces two reads (red and blue), with five nucleotides of overlap (pink). Each read produces its subgraph, and both subgraphs can be connected by their shared oligonucleotides.

The main idea behind DBG assemblers is to leverage efficient data structures to bypass any form of explicit read alignment step. DBGs are a particular type of directed string graph where each node represents an ordered set of characters with a fixed length *k*. As a directed graph, the edges of a DBG are connections from one node to another, rather than connections between nodes. In particular, the edges of a DBG must originate from a node whose last *k*−1 characters match the first *k*−1 characters of the destination node. Unlike the OLC approach, which relies on computing overlaps between reads, DBG approaches first split reads into *k*‐mers, typically between 15 and 128 bases in length. This produces a linear graph for each read. These read graphs can then be combined into a global graph, which can be traversed to uncover the original sequence. This removes the need for explicit alignment, massively reducing computational time. Once a graph is built, assembly is achieved by following a path through the graph until a stop condition is reached. Figure 7 shows an example of a simple DBG with *k* = 5. It also illustrates the inability of DBGs to resolve repeats, one of their main drawbacks. In this example, there are two loops that produce valid outputs, namely “ACGTACGTATATAT” and “GTACGTACGTACGTATATATA.”

In a real use case, *k* should be as big as possible to resolve such repeats. However, a large *k* brings its problems. One problem is that *k* must be smaller than the overlap between reads for the graph to be continuous, but this is only an issue when coverage is very low and *k* is a large fraction of the read length. The second problem is memory. Because the nodes of the DBG overlap, a genome's graph consisting exclusively of unique nodes would require at least *k* times the actual length of the genome in computer memory.

Two techniques that handle DBGs stand out in particular: hash tables (Ye et al., 2012) and Bloom filters (Bloom, 1970; Pell et al., 2012). With hash tables, each element (in this case, a *k*‐mer) is assigned a numeric value that solely depends on its content. By doing this, searching for relatively large elements in a database becomes considerably faster. This is necessary for connecting the subgraphs generated from each read to each other, which is done by checking whether a given node has already been recorded in the hash table for the whole graph. Hash tables can also be used to reduce memory requirements if handled cleverly. Another, more drastic approach to reducing memory requirements is a Bloom filter. A Bloom filter is similar to a hash table in that it relies on hashes, but unlike hash tables, Bloom filters can only be used to indicate whether an element might be present or is absent. A Bloom filter stores its data in a predefined set of bits. When adding elements to a Bloom filter, multiple hashes of that element are produced, which correspond to the indices of the bits of the Bloom filter that should be set. Checking whether an element has been added to the filter, consists of checking whether all the associated bits have been set. Because each element sets multiple bits, it is possible for all the bits associated with an element that have not been added to the filter to have been set by accident. However, this issue can be minimized through proper parametrization and the resulting structure can still be very compact.

Currently, the most commonly used DBG assembler for bacterial genomes is SPAdes (Segerman, 2020). This assembler started as a very specific assembler targeting single‐cell analysis (Bankevich et al., 2012), but throughout numerous updates, it has become a memory‐efficient and highly accurate tool. The latest version at the time of writing, 3.14, has separate approaches to handle single‐cell, isolate and metagenome data, as well as different technologies including short Illumina, Ion Torrent, ONT and PacBio reads. The key to the success of SPAdes likely lies in its origin as a single‐cell assembler. At the time when the software was developed, single‐cell sequencing required an extensive amplification step, typically multiple displacement amplification, which introduced unevenness in coverage as well as an increased rate of chimeras. SPAdes was therefore designed following the principle that it should be able to handle such inconsistencies. Moreover, unlike other DBG assemblers at the time, SPAdes used multiple *k*‐mer lengths to build a consensus graph. This was shown to be a superior approach for metagenomics (Vollmers et al., 2017). Another recent assembler operating on multiple *k*‐mers is MEGAHIT (D. Li et al., 2016). Unlike SPAdes, MEGAHIT was designed at its core as a metagenome assembler, with a focus on computational efficiency by making use of succinct DBGs (D. Li et al., 2015). This increased performance over SPAdes and other assemblers significantly when it was first released in 2015. Nowadays, these advantages are still applicable, but they are not as important. Moreover, the performance of MEGAHIT is generally worse than SPAdes and as such, it should probably be seen as a backup in case the available memory or time is insufficient to use SPAdes (Forouzan et al., 2018; van der Walt et al., 2017; Z. Wang et al., 2020).

DBG assemblers are not limited to SPAdes and MEGAHIT. Probably the earliest DBG was EULER (Pevzner et al., 2001), which has also been updated to handle short‐read sequencing data (Chaisson & Pevzner, 2008), but is not used nowadays. Other notable assemblers include IDBA‐UD (Peng et al., 2012), (Meta)Velvet(‐DL) (Liang & Sakakibara, 2021; Namiki et al., 2012; Zerbino & Birney, 2008), Ray (Meta) (Boisvert et al., 2010, 2012), MaSuRCA (Zimin et al., 2013), ABySS (Jackman et al., 2017), and SOAPdenovo2 (R. Luo, Liu, et al., 2012). For the latter three, while not explicitly designed for metagenome assembly, there may be use cases (Forouzan et al., 2018). However, they have not been updated in a while and their usage is typically not as straightforward, especially when it comes to selecting parameters.

### 3.3 Hybrid assemblers

Hybrid assembly is a term commonly used to describe a case where reads from multiple sequencing platforms are used in conjunction with each other to construct a more accurate assembly. In theory, such an approach can help to bridge the inherent drawbacks of the individual sequencers and negate some of the systematic biases. Although this terminology applies to any combination of sequencers, it refers primarily to a combination of second‐generation sequencing reads, such as those produced by Illumina sequencers, and long reads, such as those produced by ONT sequencers or PacBio sequencers. In the case of Illumina reads with ONT reads, the long reads have a high error rate, which the short reads can compensate for. Conversely, the short reads on their own are insufficient to resolve long repeats such as gene duplications, whereas long reads can. By combining both sequencing methods, one could get in theory the best of both worlds. One additional factor in this particular case is throughput, which results in long reads having lower coverage than the short reads for the same sample. Hybrid assembly with short and long reads can generally be done in one of three ways. The first approach consists of correcting long reads with short reads (read polishing), then assembling these long reads. The second approach consists of creating a preliminary assembly graph using only the short reads, then using long reads as a guide to traverse said graph, which is the strategy used by most assemblers, including HybridSPAdes (Antipov et al., 2016), OPERA‐MS (Bertrand et al., 2019), Unicycler (Wick et al., 2017) and HASLR (Haghshenas et al., 2020). Finally, the third strategy consists in assembling long reads and correcting the result using short reads. For this approach, we are not aware of individual software that can perform this task. Instead, one would rely on a long read assembler (Section 3.1) followed by a dedicated polishing tool such as pilon (Walker et al., 2014) or racon (Vaser et al., 2017), or use a combination of a read alignment tool such as Minimap2 and a sequence consensus tool such as BCFtools mpileup (Danecek et al., 2021).

In our experience, results are mixed, as current software has trouble incorporating all the input data into a cohesive whole, but as highlighted by Van Damme et al. (2021) when proposing their metagenomics pipeline called MUFFIN, a thorough review of hybrid assemblers is still pending. Moreover, publications presenting new assemblers typically show them performing very well in a limited context and with a selected number of reference genomes. They are typically tested using samples containing less than a hundred real genomes (Rinke et al., 2016; Sevim et al., 2019), a few hundred simulated genomes (Quince et al., 2017; Sczyrba et al., 2017) or real samples for which the ground truth is not available (e.g., Z. Wang et al., 2020; Wick et al., 2021). However, real‐world metagenomes are expected to be much more diverse and are estimated to contain hundreds (Power et al., 2014) to tens of thousands (Aguinaga et al., 2018) of strains per sample depending on the nature of the environment.

### 3.4 Other assemblers

Providing an exhaustive list of assemblers is out of our scope (for a review see Yang et al., 2021). We rather present the modern open‐source landscape for assemblers and their underlying principles, and do not include many older assemblers, closed‐source tools or assemblers that are exceedingly specialized. In addition, although some of the tools mentioned so far have been presented as assemblers, a large fraction of their efficacy comes from the various pre‐ and postprocessing steps that these tools perform. It used to be that read correction, assembly, scaffolding and polishing each had their dedicated software, and different tools had different priorities that could lead to suboptimal performance in some configurations. By contrast, newer assemblers, such as metaSPAdes and CANU, are designed to handle uncorrected reads as their base input, and using third‐party read correction tools is not advised (Koren et al., 2017; Nurk et al., 2017).

## 4 BINNING

While information about specific genes, such as antibiotic resistance genes, within metagenomic data may be extracted from assemblies without addition preprocessing, it is often interesting to group the genomic fragments according to taxonomic relatedness, ideally down to individual species or strains. When performed without a reference, this process is called binning and mediates reconstructing individual genomes from metagenomes, that is, MAGs. It is clear that not each bin contains the necessary information to be considered a MAG. Therefore, the minimum information about a MAG (MIMAG) standard was introduced to ensure quality (Bowers et al., 2017). The latter states that an archaeal or bacterial high‐quality MAG must be >90% complete, contain <5% contamination, and include the 23S, 16S and 5S rRNA genes, and at least 18 tRNA genes (Bowers et al., 2017). However, verifying if previously unsequenced genomes meet these requirements is itself prone to error. The reference‐based alternative to binning, that is, classification, is more closely related to identification and annotation and will be covered in the next section.

Binning is commonly performed after assembly and before annotation. At this point in the analysis, genomes are likely to be fragmented into nonoverlapping sequences called contigs. Therefore, binning is a rather complex problem, which relies almost exclusively on heuristics, since it has to use meta‐data and meta‐knowledge to group contigs. Indeed, if such a grouping would be possible without meta‐data and meta‐knowledge, this step would not have to be separate from assembly. Moreover, since the aim of metagenomics is often to detect novel organisms, this process cannot rely on references. Furthermore, even when studying relatively well‐known environments, aligning to a reference is often impossible due to the lack of a sufficiently close one and the high computational cost of searching large databases. Both result in the need to perform de novo binning.

Contig coverage and composition are typically used to guide the process, and the detection of universally conserved genes can also be used to either serve as starting points (Wu et al., 2016) or validate the results (Parks et al., 2015; Simão et al., 2015). However, this methodology is still rather error‐prone (Goussarov et al., 2022).

An alternative to binning after assembly is binning at read level. According to Kyrgyzov et al. (2020), the main advantage of binning at read level is that this process would not be affected by biases and errors introduced during the assembly process toward the more abundant species. Thus, read‐based binning, if successful, could help analyze low‐abundance species. However, two disadvantages to this approach encourage the “assembly‐first” paradigm. The first is computation cost, as sequencing data can typically be between 30 and 200 times larger than the assemblies obtained from it. The second is the sequence length. Intuitively, longer sequences are more likely to contain stretches that can uniquely link them to a given genome, whereas shorter sequences such as Illumina reads have the potential to have matches in multiple genomes.

### 4.1 Information used to perform binning

To separate genomes within a bacterial community from each other (assuming that they are fragmented), it is necessary to find a signal that is similar within closely related DNA sequences, but dissimilar to less‐related genomes. In bacterial genomes, such a signal is found in oligonucleotide frequencies, with each species having a specific set of over‐ and underrepresented oligonucleotides. This has already been observed three decades ago (Burge et al., 1992) and different methods have leveraged different approaches to make use of oligonucleotide composition to group contigs into bins. A notable example of this approach was proposed by Teeling, Meyerdierks, et al. (2004), which strengthened the notion that tetranucleotide frequencies contain a useful signal for grouping genomic fragments. Later, a similar principle was used in CompostBin (Chatterji et al., 2008), where principal component analysis was performed on the reads based on their tetranucleotide frequencies.

**Figure 8:**
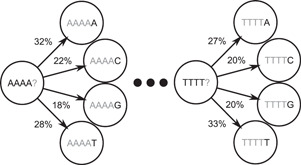
A simple Markov model is the set of transition probabilities in a text. In this case, assuming that a subsequence ends with AAAA, there is an 18% chance that the next nucleotide is guanine according to the Markov model, which describes the genome from which it was produced.

Unfortunately, an intragenomic variance of oligonucleotide frequencies is quite high when looking at fragments with a length below 10,000, which are quite common when dealing with assemblies of complex metagenomes (Forouzan et al., 2018; Kang et al., 2019; Papudeshi et al., 2017). Better performing approaches were needed and these typically involve Markov models or variants thereof. The general idea behind this is to view DNA as a sequence of nucleotides that can be inferred from the previous oligonucleotide according to a probabilistic model (Figure 8). Because Markov models are a natural choice for creating stochastic representations of genomes, they have been used in various tools, including binning tools such as SCIMM (Kelley & Salzberg, 2010) and LikelyBin (Kislyuk et al., 2009).

In addition to oligonucleotide composition, bins can also be separated by looking at universally conserved genes. These include the ribosomal RNA (rRNA) genes, and others, such as *recA*, collectively referred to as universally conserved marker genes (UCMGs) whose total number is 107 according to Ankenbrand and Keller (2016). MaxBin (Wu et al., 2014) is an example of a binning tool that utilizes UMCGs as part of its procedure, where these genes are used to initialize clusters that are then expanded using the oligonucleotide composition of contigs.

Another intuitive method to separate genomes within an assembly is to rely on coverage, which is computed by counting the total number of bases from the reads that can be aligned to the final assembly. Assuming that the relative abundances of each species within a metagenome are different from each other, the relative abundance of reads should follow a similar trend. This principle was first used in AbundanceBin (Wu & Ye, 2011) and later implemented in a variety of tools that also relied on composition, including CONCOCT (Alneberg et al., 2014), MetaBAT (Kang et al., 2015) and COCACOLA (Lu et al., 2017), to name a few. However, abundance on its own is insufficient to perform accurate binning. Indeed, sequencing depth is typically heterogeneous even within individual strains and different strains can have similar abundances. Therefore, each of the software mentioned above integrates coverage information in different ways.

Composition, universally conserved genes and coverage can be derived from any assembly without additional external information. However, a plethora of statistical and machine‐learning approaches can be used to augment and exploit these data. As an example, BMC3C uses automatic gene detection that enables codon usage analysis (Yu et al., 2018).

From the previous paragraphs, it should be clear that there are a variety of methods for separating contigs into bins. As each method has its inherent advantages and drawbacks, rather than selecting one binning tool exclusively, some developers have opted to derive a consensus binning from multiple binning tools. The latter refers to “bin refinement” and is used for instance in MetaWrap (Uritskiy et al., 2018) and DASTool (Sieber et al., 2018). However, none of these methods are particularly reliable for highly complex mocks, with bin refinement tools performing notably better than individual binning tools (Yue et al., 2020).

### 4.2 Binning using data from molecular techniques

Next to using signals within contigs, molecular techniques can also be applied to mediate binning. Currently, one of the leading approaches is exploiting 3D contact frequencies quantified by chromosome conformation capture experiments (3C, Hi‐C) (Lieberman‐Aiden et al., 2009). The approach is designed to cross‐link DNA fragments in close physical proximity to each other before sequencing, that is, links DNA from the same cell in metagenomics samples. By aligning the cross‐linked fragments to the assembly, it is possible to group contigs that originate from a given taxon more accurately (Beitel et al., 2014; Burton et al., 2014). Unfortunately, Hi‐C data have inaccuracies that require in‐depth analysis to be resolved, primarily due to spurious contacts between unrelated regions and biases in the number of cross‐links. State‐of‐the‐art tools that can effectively avoid issues arising from these inaccuracies include MetaTOR (Baudry et al., 2019), bin3C (DeMaere & Darling, 2019) and HiCBin (Du & Sun, 2022). Only MetaTOR includes the alignment step, which must otherwise be performed manually.

### 4.3 Bin validation

As binning is a rather inaccurate process, that is, bins often do not translate to MAGs, the result of the binning process should be checked. The best way to accomplish this is via reference‐based approaches, which may not always be available for the prokaryotes contained in a metagenome of interest. An alternative is to rely on UCMGs, which is most commonly done with CheckM (Parks et al., 2015) and sometimes BUSCO (Manni et al., 2021). A recent alternative to these approaches exists in the form of GUNC (Orakov et al., 2021), which is a gene‐centric approach using all genes in a genome. In addition, we have recently developed MAGISTA, which is based on machine learning and integrates data from multiple sources to address the deficiencies of the other approaches (Goussarov et al., 2022).

## 5 MEANINGFUL LABELING OF SEQUENCES

After assembly, the logical next step is to name sequences, a process that is called differently depending on the nature of what is being named and how. Broadly speaking, it can be separated into two classes: identification—the process of assigning a taxonomic name for a group of sequences, and annotation—the process of locating and naming genetic elements (primarily genes). In the case of metagenomes, identification may also be performed on individual contigs or reads. This process, called classification, can be performed in combination with or as an alternative to binning, depending on the extent to which the target environment has been characterized.

### 5.1 Identification

Identification is in most cases rather straightforward—as it consists of aligning the contigs to known reference genomes to find the best match. Despite significantly improved alignment algorithms through the use of indexing and heuristics, this step remains computationally expensive if a rigorous approach involving local alignment is used (Zielezinski et al., 2017). This becomes especially problematic as the number of references grows. Although there are currently only around 20,000 prokaryotic type strains with a sequenced genome, this number can be expected to increase considerably in the coming years (Lennon & Locey, 2020). As such, a prescreening step will likely become necessary if identification is to be achieved in a reasonable time frame. The keys to accomplishing this are to avoid the computationally expensive alignment step and to create reduced representations of databases. Two general ideas can be used to accomplish this. The first approach relies on reducing the size needed to store individual genomes by using oligonucleotide frequencies (Goussarov et al., 2020; Teeling, Waldmann, et al., 2004) or by leveraging variants of MinHash (Ondov et al., 2016; Pierce et al., 2019). The alternative is to create a carefully curated database that can be searched efficiently. This is the approach taken in Kraken (Wood et al., 2019), where the database consists of a minimally redundant subset of genomes arranged according to a phylogenetic hierarchy. By using exact string matching (see Section 6, AUTHOR CONTRIBUTIONS), it is possible to rapidly search such a database and perform identification.

### 5.2 Classification

Classification of sequences is similar to identification but inherently less accurate as it relies on highly incomplete data. As with identification, MinHash‐based methods (Ondov et al., 2019) and alignment‐based methods (Wood et al., 2019) are both applicable. Frequency‐based approaches are less suited as they are significantly affected by transposable elements, which typically do not share the oligonucleotide usage biases that are otherwise well conserved within individual genomes. As with binning, classification can be performed on both the read‐ and assembly level.

Working at the read level enables the user to estimate the relative abundance of different taxa, but is computationally expensive, sometimes prohibitively. This problem can be addressed by methods based on specific marker genes, such as MetaPhlAn2/3 (Beghini et al., 2021; Segata et al., 2012) or mOTUs2 (Milanese et al., 2019), which favor taxa detection over complete classification. Conversely, methods based on more extensive databases, such as Kraken 2 (Wood et al., 2019), Centrifuge (D. Kim et al., 2016) or CLARK (Ounit et al., 2015), are slower or require significantly more memory. The other problem with read‐based approaches is that reads can be too short to contain complete genes, which themselves are useful for classification. An example of how to solve this issue is GRASP2 (Zhong et al., 2019), which introduces a limited gene‐centric assembly step before alignment.

Contig classification is significantly less computationally demanding than read classification and may be necessary if individual reads have high error rates. It is still possible to use BLAST for this application on modern systems. More recent methods are being developed actively, including tools such as CAT (von Meijenfeldt et al., 2019) and CHEER (Shang & Sun, 2021), both of which aim to improve the accuracy of previously undiscovered genomes by bypassing the limitations of typical database‐centric approaches. For this task, contigs can also more readily be used to extract amino acid sequences, which can be used by tools such as DIAMOND (Buchfink et al., 2015) and Kaiju (Menzel et al., 2016), although these tools are also applicable at the read level.

### 5.3 Annotation

Commonly used software for gene detection and annotation are Prodigal (Hyatt et al., 2010) and Prokka (Seemann, 2014), respectively. Prodigal is popular thanks to its ability to detect previously unknown genes all the while limiting the number of false‐positive detections (Hyatt et al., 2010). It achieves this by using a “trial and error” approach in which all potential genes are first detected and scored to create a model, whose parameters are then fine‐tuned over multiple iterations. Prodigal takes into account GC codon biases, start codon biases, Shine−Dalgarno sequences and hexamer composition. It also performs analysis on potentially overlapping genes, selecting the best scoring ones to build the model. Prodigal was also partly based on expert curation with regard to the data set that was used to optimize its heuristic parameters and precautions were taken during its design to avoid “overfitting” (this term is only partially appropriate in this case). While this approach is suited for single genomes, it was subsequently adapted to work on metagenomes as well. As a result, Prodigal remained the dominant gene detection tool and has been used in a wide variety of cases, from characterizing novel environments (Tully et al., 2018) to tool validation (Nurk et al., 2017) and incorporation into new pipelines (Lin & Liao, 2016). One drawback of Prodigal is that it focuses on coding sequences, as illustrated by its reliance on codons. Therefore, genes that are not translated, such as the particularly relevant 16S ribosomal RNA gene, require other tools.

Although Prodigal is excellent at detecting genes, and more importantly, avoiding spurious detections that could occur if the genes were detected through alignment to reference data sets, it does not provide any useful information regarding their function. This is done by searching a gene database, such as KEGG (Kyoto Encyclopedia of Genes and Genomes) (Kanehisa et al., 2021), COG (Clusters of Orthologous Genes) (Galperin et al., 2021), eggNOG (Evolutionary Genealogy of Genes Non‐supervised Orthologous Groups) (Huerta‐Cepas et al., 2019) or GO (Gene Ontology) (The Gene Ontology Consortium, 2017) with an alignment tool. Even in cases where the exact sequence is not found due to errors or mutations compared to the closest reference, alignment algorithms are designed to identify homologous genes. Each database has typically its search engine and pipelines such as Integrated Microbial Genomes & Microbiomes (IMG/M) (Chen et al., 2021) and MG‐RAST (Keegan et al., 2016) integrate them into their workflow. The Prokka tool does also integrate both steps (Seemann, 2014).

## 6 ALIGNMENT

Many (sub)steps in the described metagenomics workflow rely on alignment, that is, the process of finding similar subsequences within sets of sequences and identifying the differences. Therefore, for the sake of completeness, alignment concepts were included in this overview.

Conceptually, alignment can be seen as a number of string matching operations, which can be exact or approximate. Exact string matching is useful for locating short sequences and its results can be used as a starting point for approximate string matching of larger strings. Exact string matching is generally not relevant for longer sequences, either due to the presence of sequencing or assembly errors or to a biological divergence between the query and reference sequences. However, even with these changes, homologous sequences can still be found using inexact string matching. For this case, there are essentially two ways of aligning sequences to one another: either to identify all the changes that need to occur to convert one sequence into another (global alignment) or to find the best matching regions of the two sequences (local alignment).

The distinction between global and local alignment is particularly relevant in genomes, as both types of alignment are used, but in different contexts. However, on the whole‐genome sequence scale, alignment must also handle transpositions, where a long sequence is located in one place in one genome and in another place in the other, and inversions, where sequences are replaced by their reversed complementary sequence. For this task, it is necessary to first identify orthologous sequences, a process that is severely complicated by the fact that there are usually differences between these sequences and that the abundance of these differences varies from pair to pair.

### 6.1 Exact string matching

String matching is the process of finding a given set of ordered characters (a string) of length *m*, such as an oligonucleotide, within a larger set of characters of length *n*, such as a genome. The “naïve” approach to finding similar strings is simply to check every possible position within the larger set of characters, then verify if the subsequent characters match the query. However, this approach is extremely inefficient and more efficient approaches have been developed, culminating in suffix trees stored as suffix arrays.

**Figure 9:**
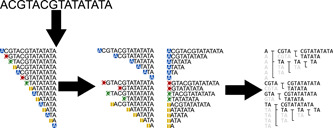
A suffix tree is a data structure containing all the possible suffixes in a chain of characters. Namely, suffixes that share the same prefix are considered to be part of the same branch, diverging into separate branches when the prefixes no longer match.

Conceptually, suffix trees are hierarchical structures containing every single possible suffix within the large set of characters it represents, including the full one (Figure 9). Such a data structure allows checking whether or not any given subsequence is contained within the full sequence in *O*(*m*) (Big O notation), rather than the *O*(*m *× *n*) of the naïve method. Construction and required memory are both *O*(*n* ^2^) (Figure 9), which rapidly becomes impractical for biological data. To resolve this issue, suffix arrays are used. Unlike explicit suffix trees, suffix arrays can not only be used to obtain the first position of any given query in *O*(*m*) time, they can also be constructed in *O*(*n*) time and occupy *O*(*n*) memory (Skiena, 2020). By using suffix arrays, it therefore becomes possible to rapidly and efficiently index genomes of any size, and then perform multiple searches in minimal time.

However, the suffix array approach still requires *O*(*n*) time to be built. While this is useful for performing multiple searches on the same genome, when searching for a single sequence within a larger sequence only once, an alternative approach exists that requires less than *O*(*n*) time in a usual case and *O*(*n*/*m*) in the best case, called the Boyer−Moore algorithm. In this approach, the query string is first used to construct a table that contains the relative location of the next identical character for each possible character. Once this array is built, it becomes possible to skip portions of the text whose ending does not match that of the query, since a mismatch can be used to infer the closest possible position of a potential match, and avoid comparing the query against all intermediate positions. Because this algorithm indexes the query rather than the larger sequence, suffix trees are generally more practical.

Unlike the ideal case of searching exact matches, for which objective and fast methods have been presented above, alignment of genetic sequences typically involves mismatches. This can be because of inherent mismatches in sequences that occur when searching for known genes in novel organisms, or when attempting to find overlaps between reads of sequencing data that contain errors. In either case, approximate string matching algorithms become necessary.

### 6.2 Global alignment

Identifying differences between two sequences is a relatively straightforward process that usually relies on edit distances, the most well‐known of which is the Levenshtein distance, which assigns an error cost for each insertion, deletion and substitution between two strings of text, and reports the sum of produced mismatches between two strings as the distance between them. Under specific conditions, these edit distances can be calculated in *O*(*s *× min(*m*,*n*)) computational time and space (Ukkonen, 1985), where *s* is a value that must be set according to how similar the sequences (of length *m* and *n*) are expected to be. This means that for sequences that are expected to be similar, distance can be computed at a low computational cost.

Because global alignment can be viewed as an optimization problem, the result of such an operation is intrinsically linked to the evaluation criteria that are sought to be optimized by the algorithm. Because of this, it is important to remember the context in which such alignments are performed. In the present work, two such contexts are particularly relevant. The first relevant context is assembly, since long‐read sequencers tend to produce more insertions and/or deletions than their short‐read counterparts (Sacristán‐Horcajada et al., 2021). Alignment is used in the OLC approach that is most effective for such reads and global alignment is relevant for the consensus step. The second relevant context is comparing genes from different strains, where changes represent mutations that occurred over long periods. In this case, insertions and deletions of single nucleotides are a lot rarer than with sequencing since they lead to frameshifting, which has the potential to render a gene inoperative. As a result, the cost associated with each type of mismatch may need to be adapted depending on the situation, though default values are typically specified in published software that is known to perform adequately for the task for which the software was intended.

An important extension or global alignment is MSA. Unlike pair‐wise sequence alignment, for which an optimal alignment (given specific evaluation criteria) can be computed within a reasonable time frame of *O*(*m *× *n*) in the worst case, the computational cost of finding an optimal solution to an MSA problem is factorial in the number of sequences. This means that the only realistic options are to rely on heuristics with no guarantees of producing an optimal solution. MSA is necessary for establishing the consensus sequences, be it for establishing the “true” sequence of the genome underlying a set of sequencing reads, or to find the common ancestor of a set of orthologous genes. For global alignment, MUSCLE (Edgar, 2004), Clustal ω (Sievers & Higgins, 2014), and the NAST algorithm implemented in the Mothur environment (Schloss et al., 2009) are all software of some renown.

### 6.3 Local alignment

Unlike global alignment, where the focus lies in uncovering the “least expensive” set of changes necessary to convert one sequence to another, local alignment focuses on locating the position of matching (sub)sequences. The most common local alignment approach is the seed and extend approach, which operates by identifying short matching subsequences within the sequences to be compared and then attempts to extend them until a significant enough number of inconsistencies is detected or one of the two sequences ends. The way these shorter sequences are identified varies from program to program.

Some programs, such as the first incarnation of BLAST (Altschul et al., 1990), rely on matching relatively short oligonucleotides of length 8−12. Their primary advantage is that a pair of short oligonucleotides obtained from two matching sequences is less likely to contain mismatches by being shorter, even if the overarching sequences have mismatches, enabling the use of exact string matching approaches to find them. One way to use short oligonucleotides is to build a table containing every location of each possible oligonucleotide. By having such a table, it becomes possible to look up the positions of any oligonucleotide without actually performing a search, which drastically reduces the time required to identify all potential matches.

Another approach is to rely on suffix trees rather than on oligonucleotides of fixed length to identify the seeds. Suffix trees can rapidly be compared to each other to identify so‐called maximal unique matches or MUMs. This can be done by merging both trees all the while keeping labels of the origin of each branch and simply finding those branches of the merged tree that have exactly two child branches of different origin (Delcher et al., 1999).

Once a seed has been established, the extension procedure can begin. This step is typically based on heuristic rules encoded as some sort of model graph based on the target sequence. One of the most well‐known software applied for the alignment of both short and long sequences is BLAST, which was first published in 1990 (Altschul et al., 1990) and adapted to a variety of cases over the years (Altschul et al., 1997; Kent, 2002; Zhang et al., 2000). As its name suggests, BLAST performs “local” alignment, which means it attempts to find conserved subsequences rather than attempting to align entire sequences against each other. BLAST uses a variant of seed and extend algorithm where short matching sequences are identified—which is done efficiently by limiting the search to closely related sequences of identical length, indexed using a fast data structure—and then extended according to empirically defined alignment scoring rules. The alignments that meet user‐specified criteria are then sorted according to their score and reported.

Another tool to perform local alignment is HMMER (Eddy, 2011), which uses profile hidden Markov models (HMMs). Compared to BLAST, HMMER is slower if simply used to align sequences. However, because it relies on a model, this means that it can assign different weights to different positions, depending on prior knowledge regarding sequences. As a result, HMMER can be considerably more useful than BLAST for studying sequence homology between organisms and relating it to evolutionary distances.

This comparison illustrates the need for different tools depending on the reason why alignment is performed. Indeed, BLAST is more useful than HMMER when attempting to discover which sequence is being studied, whereas HMMER is useful for comparing sequences from different organisms. While BLAST focuses on locating similar sequences, there is also software that can be used to evaluate how sequences are arranged. Foremost among these is Minimap2 (H. Li, 2018). This tool locates short (~20) sequences, called minimizers, which are locally minimal in the alphabetic sense (e.g., “AA” < “AB”), and matches their location between the target sequences. By using minimizers rather than the full alignment, the whole process is greatly accelerated and large‐scale modifications of DNA, such as relocations, transpositions, and large insertions and deletions (together referred to as indels) become apparent.

Alignment is computationally expensive but can be accelerated through proper indexing of sequences. Examples of indexing techniques include suffix trees for exact matches of variable length, implemented in MUMmer, and the Burrow−Wheeler transform (BWT) implemented in Bowtie (Langmead et al., 2009) and BWA (H. Li & Durbin, 2010). MUMmer (Kurtz et al., 2004) is an example of alignment based on MUMs. Unlike BLAST, which relies on oligonucleotides of a fixed length to create seeds, MUMs can be of any length. Efficient localization of MUMs is achieved using suffix trees, a data structure that stores all possible suffixes in a sequence, with all suffixes sharing a prefix being located on the same branch. This kind of data structure enables searching for exact matches in linear time, but requires a large amount of memory and only produces one match location for any sequence. However, both of these issues were addressed in the latest version of MUMmer, which has drastically reduced memory requirements for storing suffix trees and has been extended to be able to report nonunique matches (Marçais et al., 2018). Finally, if one wishes to compare multiple bacterial strains visually at a low computational cost, a software of interest is Mauve (Darling et al., 2010), which performs MSA on the scale of whole genomes and has an intuitive user interface.

### 6.4 Alignment in metagenomics

As stated previously, alignment is necessary at multiple stages in metagenome analysis. Of these, perhaps the most critical is read alignment. Reads can be aligned against a reference to identify extant taxa, against a complete assembly to estimate abundance, against individual bins to separate the sequencing data according to the genome of origin, or against longer reads to reduce error rate (read polishing), although the latter is not strictly unique to metagenomics.

Unlike alignment of assembled sequences against a reference, nonspecialized tools have an insufficient performance to perform read alignment. BWA and Bowtie2 have long been established as standard tools for aligning reads against a known reference or an assembly, but are intended for short highly accurate reads produced by Illumina and older short‐read technologies. With the advent of long error‐prone reads, newer approaches such as Minimap2 had to be developed to perform the same operation on long reads. Moreover, for metagenomics specifically, these tools are not sufficient to extract meaningful information on their own, nor are they intended to align reads against large databases. Because of this, when aligning reads against a reference database, the tools mentioned in Section 5.2 are necessary.

## AUTHOR CONTRIBUTIONS

**Gleb Goussarov**: Conceptualization (lead); writing—original draft (lead); writing—review and editing (equal); **Mohamed Mysara:** Conceptualization (supporting); writing—review and editing (supporting); **Peter Vandamme:** Conceptualization (supporting); writing—review and editing (supporting); **Rob Van Houdt:** Conceptualization (supporting); writing—original draft (supporting); writing—review and editing (equal).

## CONFLICT OF INTEREST

None declared.

## ETHICS STATEMENT

None required.

## Data Availability

Not applicable.
